# Editorial

**Published:** 2012-02

**Authors:** AJ Brink

**Affiliations:** Cardiovascular Journal of Africa

The *Cardiovascular Journal of Africa* (CVJA) is increasingly gaining recognition as a medical science journal of standing. It is widely distributed and indexed, with at least 3 000 full-text articles downloaded a month via PubMed alone. This is apart from South African and African tertiary institutions, which have access to full-text capabilities on the SABINET system.

The CVJA is now an important source of information in Africa on diseases related to the cardiovascular system. It also presents significant advances worldwide in heart disease and related technology.

It is envisaged that 10 issues of CVJA will be published in 2012; essentially a 40% expansion in journal content. This is in response to the increase in numbers of submitted and accepted manuscripts, despite the relatively high rejection rate of 50%. This will significantly shorten the time between acceptance of articles and publication.

The main obstacle to the journal’s further development is adequate funding, outside of advertising revenue. Government will be approached, possibly through the MRC, as well as international philanthropic organisations such as the Bill Gates Foundation and the Qatar Sovereign Fund. Sponsorships are constantly being sought.

Funding for medical research in South Africa and Africa is raised in a number of ways, such as from governments, international philanthropic and research organisations, sponsorship from private enterprises, societies’ membership subscriptions, campaigns to collect money from the public for heart and cancer research, and individual bequests. Whatever the amounts raised they always seem insufficient to meet the demand.

Medical research is of little value if it is not published and made available to the global community. Unpublished medial research is a waste of money, time and effort. More support is therefore needed for journal publications.

Payment to process manuscripts is now required by many journals. The CVJA has recently introduced a modest processing fee of $50 per article. In order to obtain additional funding, the journal also provides an up-to-date news section for clinicians, including conference coverage and interviews with medical experts. Additional charges for advance online publication on PubMed are now an accepted feature, which works well for those authors requiring speedy publication of results.

The splintering of medical disciplines into minor groups is a complicating factor for the publication of relevant medical research. Each group produces a journal for its internal communication and consequently, many more ‘overnight’ or ‘passingthrough journals’ come to life. ‘Birth control’ may need to be practiced by the various disciplines and all will have to consider consolidating and working together.

Good-quality research published in the CVJA is now recognised, with the introduction of the Andries Brink-Kaye Award. In 2010 this award was presented to Andrea de Kock of Potchefstroom University for her article ‘Coping and metabolic syndrome indicators in urban black South African men: the SABPA study’ in *Cardiovasc J Afr* 2010; 21(5): 268–273. This concept of coping with stress has not to our knowledge been studied before in African men. The award for 2011 is now under consideration by the CVJA evaluating panel.

There is little doubt that the CVJA has filled a need and has a promising future. We will be celebrating 25 years of publishing cardiovascular research in Africa in 2014, which is only 24 months away. As part of our strategy, we are planning an African celebratory issue. Relevant ideas from our readers and other stakeholders would be appreciated.

